# Atlanta Medical College

**Published:** 1884-03

**Authors:** 


### ATLANTA MEDICAL COLLEGE.

The Commencement exercises of the Atlanta Medical College were held on Thursday evening, February 28th, at DeGive's Opera House, in the presence of a large and cultured audience.

There were forty-eight graduates as follows :

J. B. Adams, Alabama; B. W. Allen, Georgia; W. C. Ashley, Georgia; J. A. Avant, Alabama; R. E. L. Barnum, Georgia; W. T. Carter, Georgia; W. G. Lewis, Georgia; W. J. Mason, Alabama J. D. McCollum, Georgia; J. I. McConnell, Georgia; W. F. McCurdy, Georgia; F. K. Mitchell, Georgia; W. J. Clements, Georgia; A. B. Cole, Georgia; Frank Cordle, Georgia; J. M Crawford, Georgia; S. A. Crumbly, Georgia; Jeff. Davis, Georgia; A C. Moreland, Georgia; B. C. Morgan, Alabama; S. A. Murray, Georgia; J. A. Parsons, Georgia; J. B. Powers, Georgia; B. W. Pirkle, Georgia; D. W. Dorsett, Georgia; F. J. Erwin, Georgia; R. C. Folger, South Carolina; T. J. Garner, Arkansas; G. H. Goldsmith, Georgia; L C. Goneke, Georgia; A. M. Raines, Georgia; H. L. Richardson, Texas; A. Scott, Alabama; E. N. Shaw, Georgia; W. T Spears, Georgia; J. S. Tankersley, Georgia; J. G. Greene, Georgia; D. J. Heron, Alabama; T. B. Hollis, Georgia; J. W. Hurt, Georgia; E. R. Kennebrew, Georgia; William Legue, Netherland; W. C. Thomason, South Carolina; A. P. Thornton, Texas; R. P. White, Georgia; A. W. Wilder, Arkansas; L B. V. Woolley, Georgia; W. D. Wright, Georgia.

The first, second and third honor prizes were awarded Dr. Alva W. Wilder, of Arkansas; Dr. Thomas B. Hollis, of Georgia, and Dr. L. B. V. Woolley, of Georgia.

The Faculty prizes were awarded as follows :

Dr. W. F. Westmoreland's to Dr. J. A. Parsons; Dr. W. A. Love's to Dr. J. W. Hurt; Dr. V. H. Taliaferro's to Dr. A. C. Moreland; Dr. A. W. Calhoun's to Dr. L. B. V. Woolley; Dr. J. H. Logan's to Dr. W. T. Spears; Dr. J. S. Todd's to Drs. Woolley and Shaw; Dr. J. A. Gray's to Dr. T. B. Hollis.

